# Endometrial E-cadherin and N-cadherin Expression during the Mid-Secretory Phase of Women with Ovarian Endometrioma or Uterine Fibroids

**DOI:** 10.3390/jpm14090920

**Published:** 2024-08-29

**Authors:** Bo Seong Yun, Na Yeon Yun, Jung Eun Lee, Minyeon Go, Hee Yeon Jang, Ji Eun Park, Ju-Won Roh, Sung Shin Shim

**Affiliations:** 1 Department of Obstetrics and Gynecology, CHA Ilsan Medical Center, CHA University, Goyang 10414, Republic of Korea; bosungyun@chamc.co.kr; 2 Department of Biomedical Science, College of Life Science, CHA University, Seongnam 13488, Republic of Korea; skdusxx@chauniv.ac.kr (N.Y.Y.); 1228wjddms@chauniv.ac.kr (J.E.L.); 3 Center for Genome Diagnostics, CHA Biotech Inc., Seoul 06125, Republic of Korea; raculase@chamc.co.kr (M.G.); jhyeon@chamc.co.kr (H.Y.J.); ditto6626@chamc.co.kr (J.E.P.); 4 Department of Obstetrics and Gynecology, CHA Gangnam Medical Center, CHA University, Seoul 06125, Republic of Korea

**Keywords:** ovarian endometrioma, uterine fibroid, E-cadherin, N-cadherin, endometrial receptivity, secretory phase, infertility

## Abstract

Background: Endometriosis and uterine fibroids are benign conditions frequently linked to subfertility/infertility. Recent research has highlighted the importance of epithelial–mesenchymal transition between embryonic and endometrial cells in the context of embryo implantation. Additionally, the adverse endometrial environment during implantation has been proposed as a mechanism contributing to infertility in endometriosis. Nevertheless, the role of cadherin molecule alterations in relation to endometrial receptivity and embryo invasion remains a subject of controversy. Methods: We investigated the expression patterns of E-cadherin and N-cadherin in the endometria of women with ovarian endometrioma or uterine fibroids and assessed whether they differed from those of healthy women. We enrolled 17 women with ovarian endometrioma, 16 with uterine fibroids, and 6 healthy women. Endometrial tissues were obtained at the mid-secretory phase on days 19–24 of the menstrual cycle. The E-cadherin and N-cadherin mRNA and protein expression levels were measured using quantitative reverse transcriptase polymerase chain reaction and Western blot analysis, respectively. Results: The E-cadherin and N-cadherin mRNA expression levels were higher and lower, respectively, in the endometrium of women with ovarian endometrioma than in those of the controls. In the endometrium of women with uterine fibroids, similar patterns with higher E-cadherin and lower N-cadherin levels were observed compared with that of the controls. Protein expression showed similar patterns. Conclusions: Our findings revealed higher E-cadherin expression and lower N-cadherin expression in the endometria of women with infertility-related diseases than in those of healthy women in the mid-secretory phase. This suggests a resistance to endometrial receptivity, potentially reflecting mesenchymal–epithelial transition properties.

## 1. Introduction

Endometriosis is a representative infertility-related disease in women. Uterine fibroids are also considered a possible cause of infertility. The prevalence of endometriosis is 20–50% in women with subfertility [1], while the prevalence of uterine fibroids seems to be considerably lower than that of endometriosis in women with infertility; however, they still occur in 5–10% of women with infertility [2].

The pathogenesis of reduced fertility rates in women with endometriosis and uterine fibroids varies. Endometriosis causes disturbed folliculogenesis and chronic inflammatory reactions in the pelvic peritoneum, leading to infertility [3]. Further, an unfavorable environment for implantation has recently emerged as a significant mechanism of infertility, especially in women with endometriosis, where the implantation rate is notably reduced during natural cycles or assisted reproductive technologies (ART) treatments [4]. This condition is associated with altered expression patterns of implantation genes or epigenetic factors in the eutopic endometrium [5,6,7,8]. The mechanism of infertility in uterine fibroids involves decreased blood supply, increased contraction of the uterus, hormonal changes, and physical effects such as endometrial deformation [9]. In terms of endometrial distortion, fibroids that create irregularities in the uterine cavity are linked to reduced fertility rates [10]. However, there are also reports suggesting that fibroids which do not distort the uterine cavity can still negatively impact ART outcomes [11,12].

Implantation requires a precise and synchronized interaction between the embryo and the endometrium during the mid-secretory phase, characterized by “endometrial receptivity to implantation” [13]. This period is referred to as the window of implantation (WOI). Luteal phase deficiency during this period can impact both infertility and recurrent pregnancy loss [14]. This is not only due to insufficient progesterone production or a shortened duration but also because, even with adequate progesterone levels, inadequate endometrial response or progesterone resistance, such as dysfunction in progesterone receptors, can prevent the formation of an optimal WOI [15]. Additionally, regulating an optimal WOI for implantation involves several important factors, including suitable synchrony between endometrial cells, proper synchrony between the endometrium and the embryo, typical morphological characteristics of endometrial glands, and potential silent genetic variations [16]. The representative changes in the endometrial markers involved in endometrial receptivity have been reported in infertility-related disease. In endometriosis, aberrant expression of endometrial genes, including homeobox (Hox) genes, integrins, and leukemia inhibitory factor (*LIF*), affects endometrial receptivity [5,6,7]. Uterine fibroids also showed decreased expression of *HOXA10*, *HOXA11*, and *LIF*, which are essential for implantation [17,18].

As the process of implantation requires apposition, adhesion, and invasion, the functional integrity controlled by adhesion molecules appears to influence the embryo’s capacity to implant concerning endometrial receptivity [19]. In recent studies, the significance of the epithelial–mesenchymal transition (EMT) process between embryonic cells and endometrial cells in embryo implantation has been emphasized [20]. Abnormalities in EMT within endometrial cells can lead to implantation failure. Therefore, it is important to investigate whether the characteristic loss of cell–cell adhesion associated with EMT induction is observed in the endometria of patients with infertility-related diseases.

Cadherins are junctional complex proteins involved in cell–cell adhesion. Epithelial cadherin (E-cad) plays a crucial role in preserving the integrity and functionality of epithelial tissues, whereas neural cadherin (N-cad) in epithelial cells is involved in EMT [21]. Some animal studies have reported a reduction in E-cad levels in endometrial epithelial cells where embryo implantation occurs [22,23], and a decrease in both E-cad and N-cad expression at the time of implantation has also been reported [24]. If cadherin distribution in the endometrium can affect embryo implantation during the WOI, it is necessary to examine the changes in cadherin expression in the endometria of women affected by infertility-related conditions such as endometriosis and uterine fibroids.

Therefore, in this study, we aimed to investigate the mRNA and protein expression patterns of E-cad and N-cad in the endometria of women with ovarian endometrioma or uterine fibroids and whether they differ from those of healthy women.

## 2. Materials and Methods

### 2.1. Study Population and Sample Collection

This prospective cohort study included women diagnosed with ovarian endometrioma or uterine fibroids at the Department of Obstetrics and Gynecology of CHA Ilsan Medical Center, CHA University, between April 2022 and March 2023. This study included women aged ≥20 and ≤45 years with regular menstruation cycles between 25 and 30 days who were diagnosed with ovarian endometrioma or uterine fibroids based on ultrasound or magnetic resonance imaging and who participated by voluntary agreement. The study excluded women who met any of the following criteria: absence of a uterus due to prior surgery, lack of coital history, receipt of hormonal treatment within the past 3 months, presence of coagulation disorders, untreated thyroid gland disease within the past 3 months, antiphospholipid syndrome, or conditions such as adenomyosis, hydrosalpinx, or chronic endometritis. In addition, women with both ovarian endometriomas and uterine fibroids were excluded due to the difficulty of determining the effects of each condition. However, if a woman had an ovarian endometrioma and a uterine fibroid smaller than 3 cm that was not of the submucosal type, the case was categorized into the ovarian endometrioma group. Women without endometrioma or uterine fibroids based on the imaging tests were enrolled in the control group. The study protocol was approved by the Institutional Review Board and Ethics Committee of CHA Ilsan Medical Center, CHA University (IRB No. 2022-02-001), and all participants provided informed consent.

We recorded age, body mass index, nulliparity, and numeric rating score of dysmenorrhea-related pain from all subjects. Serum CA125 level and anti-Müllerian hormone level were also obtained from subjects with ovarian endometrioma or uterine fibroids. For the ovarian endometrioma group, clinical data were collected on tumor size, laterality (unilateral or bilateral), and the presence of uterine fibroids smaller than 3 cm. In the uterine fibroids group, detailed information was obtained regarding tumor size, number, and type (subserosal, intramural, or submucosal) of the fibroids.

Endometrial tissues were obtained at the mid-secretory phase during days 19–24 of the menstrual cycle by biopsies using an endometrial suction catheter (Pipelle; Laboratoire CCD, Paris, France). Each sample was divided into two parts and placed in a cryotube. The first one was immediately collected in RNAlater (Thermo Fisher Scientific, Inc., Waltham, MA, USA), stored at 4 °C overnight with the supernatant removed, and then moved to a deep-freezer (−80 °C). The second one was immediately stored at −80 °C without any processing.

### 2.2. RNA Extraction and Quantitative Reverse Transcriptase Polymerase Chain Reaction (RT-PCR)

Total RNA was extracted from endometrial tissues using Accuprep^®^ Universal RNA Extraction Kit (Bioneer, Daejeon, Republic of Korea) following the manufacturer’s protocol. RNA purity was assessed using the optical density at 260 and 280 nm wavelengths. The first DNA strand (cDNA) was synthesized from total RNA (500 ng) using the Oligo(dT)18 Primer (Thermo Scientific, Waltham, MA, USA), dNTP Mix (Promega, Madison, WI, USA), RNaseOUT™ Recombinant Ribonuclease Inhibitor (Invitrogen, Carlsbad, CA, USA), and RevertAid Reverse Transcriptase (Thermo Scientific, Waltham, MA, USA). Quantitative RT-PCR was performed using FastStart SYBR Green Master Mix (Roche Diagnostics, Mannheim, Germany) and the CFX Duet Real-Time PCR System (Bio-Rad, Hercules, CA, USA). The primers used in this study are listed in Table 1. The thermal-cycling conditions were denaturation for 10 min at 95 °C followed by 40 cycles of denaturation for 15 s at 95 °C, annealing for 1 min at 60 °C, and extension for 1 min at 72 °C. Relative gene expression levels were calculated with the $2^{-∆∆Ct}$ method, with β-actin used as the internal control.

### 2.3. Protein Extraction and Western Blot Analysis

Proteins were extracted from endometrial tissues using Pierce RIPA buffer (Thermo Scientific, Waltham, MA, USA) with a complete Mini Protease Inhibitor Cocktail (Roche Diagnostics, Mannheim, Germany). Protein concentrations were quantified using a Pierce™ BCA Protein assay kit (Thermo Scientific, Waltham, MA, USA). Afterward, the protein solution was added with 5× loading buffer (Biosesang, Gyeonggi-do, Republic of Korea) and boiled at 95 °C for 10 min for denaturation. The pooled samples were loaded onto 10% sodium dodecyl-sulfate polyacrylamide gel electrophoresis gels and transferred onto polyvinylidenefluoride membranes (Bio-Rad, Hercules, CA, USA). Membranes were blocked with 5% skimmed milk or bovine serum albumin in Tris-buffered saline containing Tween 20 at room temperature and incubated with primary E-cad (1:1000, Cell Signaling Technology, Danvers, MA, USA) and N-cad (1:1000, Cell Signaling Technology, Danvers, MA, USA) antibodies overnight at 4 °C. GAPDH (1:5000, Invitrogen, Carlsbad, CA, USA) antibody was used as the internal control for normalization. The membranes were subsequently incubated with horseradish peroxidase-conjugated secondary and goat anti-rabbit or goat anti-mouse antibodies at room temperature for 1 h, and the blots were visualized using enhanced chemiluminescence detection reagent (Bio-Rad, Hercules, CA, USA). For expression analysis, the Image Lab Touch 6.1 Software (Bio-Rad Laboratories, Hercules, CA, USA) was used. Protein expression values were derived as expression of target protein/expression of GAPDH protein.

### 2.4. Statistical Analyses

Values are presented as medians and interquartile ranges (IQRs). For quantitative RT-PCR, normality was calculated using the Shapiro–Wilk test. For comparisons between the two groups, a two-sided Mann–Whitney U test was used. Depending on the normality tests, a two-sided *t*-test was used for Western blot data to determine the statistical significance of differences between groups. For categorical variables, both the chi-square test and Fisher’s exact test were used, as appropriate. These assays were performed using R studio software (v4.1.0). *p* < 0.05 was considered significant. All experiments were repeated.

## 3. Results

Seventeen women with ovarian endometrioma and sixteen with uterine fibroids were enrolled. As a control, the endometrial tissue samples of six healthy women were obtained. The patient characteristics are listed in Table 2. There were no significant differences in the median age, BMI, and NRS for dysmenorrhea-related pain between the control group and the group with ovarian endometrioma or uterine fibroids, respectively. Two out of six (33.3%) healthy women were nulliparous, which was not significantly different when compared to the groups with ovarian endometrioma or uterine fibroids.

**Figure 1:**
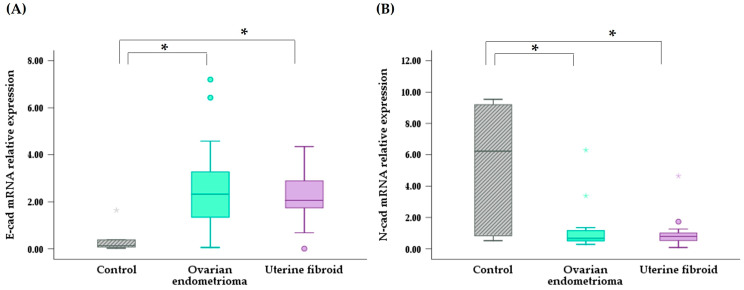
Relative mRNA expression of E-cad (**A**) and N-cad (**B**) in the endometria of women with ovarian endometrioma or uterine fibroids. Boxes represent median and IQR values, whiskers represent maximum and minimum values, and colored dots or stars represent the outlier values. E-cad, E-cadherin; N-cad, N-cadherin. * *p* < 0.05.

Figure 1 shows the relative mRNA expression of E-cad and N-cad. The median value of E-cad mRNA expression in the endometria of patients with ovarian endometrioma was significantly higher than that in the endometria of the controls (2.32; IQR 1.13–3.79 vs. 0.14; IQR 0.07–0.70, *p* = 0.002). Similarly, the median value of E-cad mRNA expression in the endometrium of patients with uterine fibroids was also significantly higher than that in the endometria of the controls (2.06; IQR 1.71–2.96 vs. 0.14; IQR 0.07–0.70, *p* = 0.002). In contrast, the median value of N-cad mRNA expression in the endometria of patients with ovarian endometrioma was significantly lower than that in the endometria of the controls (0.67; IQR 0.50–1.23 vs. 6.23; IQR 0.75–9.29, *p* = 0.024). Similarly, the median value of N-cad mRNA expression in the endometria of patients with uterine fibroids was also significantly lower than that in the endometria of the controls (0.79; IQR 0.53–1.03 vs. 6.23; IQR 0.75–9.29, *p* = 0.040).

**Figure 2:**
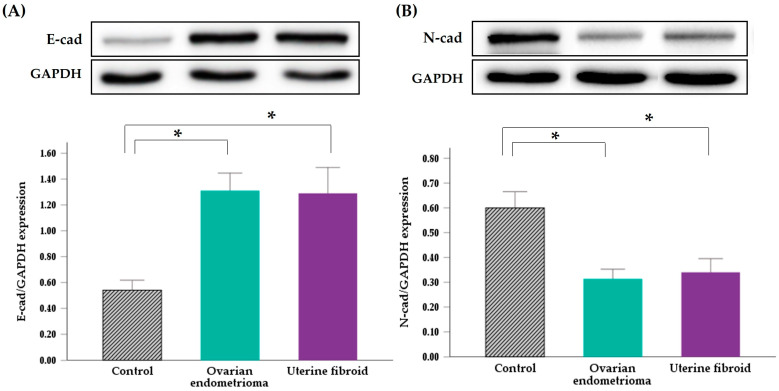
Representative Western blot images (**top**) and bar graphs (**bottom**) representing summary densitometric data showing protein expression of E-cad (**A**) and N-cad (**B**) in the endometria of women with ovarian endometrioma or uterine fibroids. Bar and whisker represent mean and SD values. E-cad, E-cadherin; N-cad, N-cadherin. * *p* < 0.05.

Figure 2 shows the protein expression levels of E-cad and N-cad determined by Western blot analysis. The protein expression of both E-cad and N-cad showed similar patterns to the mRNA expression in the endometria of patients with ovarian endometrioma or uterine fibroids. The relative expression of E-cad protein was high, being 2.52- and 2.55-fold higher than that of the controls (*p* = 0.013, *p* = 0.049), while the relative expression of N-cad protein was low, being 0.55- and 0.59-fold higher than that of the controls in the endometria of patients with ovarian endometrioma and fibroids, respectively (*p* = 0.030, *p* = 0.043).

## 4. Discussion

In this study, we observed a higher E-cad expression and a lower N-cad expression in the endometria of women with infertility-related diseases, including endometriosis and uterine fibroids, compared with those of healthy women in the mid-secretory phase. This suggests that alteration of these adhesion molecules during the WOI may be associated with infertility-related diseases through resistance to endometrial receptivity.

For successful implantation, the endometrium must be in its most receptive state. The luminal epithelium maintains the solid integrity of the endometrium through the junctional complex and undergoes cyclic remodeling under hormonal influence [25]. Consequently, the endometrium typically resists embryo implantation outside the WOI throughout the menstrual cycle. However, in vitro studies indicate that when the endometrium is compromised, embryos can implant readily even without prior hormonal priming [22]. This suggests that a change in the integrity of the endometrium can improve the receptivity of the endometrium and make implantation easier.

Cadherins comprise a superfamily of 114 calcium-dependent membrane proteins that regulate cell–cell adhesion [26]. E-cad maintains the epithelial phenotype and regulates tissue homeostasis by influencing diverse signaling pathways [27]. Therefore, down-regulation of E-cad is frequently observed in malignant epithelial cancers and contributes to metastatic spread [28]. In contrast, N-cad is predominantly expressed in non-epithelial tissues and usually serves as a marker for the ongoing EMT [21]. During EMT, there is an up-regulation of N-cad and a down-regulation of E-cad, marking the initial stages of the process [29]. Epithelial cells undergo EMT, transitioning to gain mesenchymal characteristics subsequent to the dissolution of intercellular junctions [30]. This EMT process has also been suggested as a mechanism involved in implantation because epithelial cells are a strong barrier to embryo implantation that need to be breached. An in vitro model has demonstrated that JAr spheroids, which resemble pre-implantation human embryos, stimulate migration and induce the expression of markers associated with EMT in endometrial epithelial cells [31]. E-cad and N-cad are related to the EMT process, and these alterations in cell–cell connections could induce an environment for easy implantation.

Our study revealed a low E-cadherin and a high N-cadherin expression in healthy women, indicating that the endometrium may be prepared for the EMT process and is more receptive to implantation in the mid-secretory phase. Conversely, the endometria of women with endometrioma or uterine fibroids showed a higher E-cad and lower N-cad expression than those of healthy women in the mid-secretory phase, suggesting that this low EMT capacity and inappropriate receptivity are related to low fertility.

Tiwari et al. also demonstrated that in vitro using mouse cells, there was a reduction in E-cad expression accompanied by elevated levels of N-cad and SNAIL expression in a typical hormonal cycle, indicating the presence of EMT in the endometrial luminal epithelium during embryo implantation [24]. In a human study, E-cad and total β-catenin protein expression were notably elevated in both luminal and glandular epithelial cells in patients experiencing infertility due to endometriosis or unexplained infertility compared to healthy fertile controls during the mid-secretory phase, which closely aligns with our findings [32]. Recently, Huang et al. also found that E-cad was increased and vimentin was reduced in the endometria of women with endometriosis, suggesting that EMT was blocked during mid-secretory transformation in endometriosis [33]. However, another investigation reported the opposite results. Makker et al. found that infertile women with intramural fibroids exhibited notably decreased levels of E-cad mRNA and protein, while N-cad and β-catenin levels showed no significant changes [34]. Additionally, Bi et al. demonstrated in both in vitro and mouse in vivo studies that HOXA10 binds to the E-cad promoter region, directly regulating its expression and enhancing endometrial receptivity, thereby increasing embryo adhesion and implantation [35]. However, this finding does not necessarily extend to the endometria of women with endometriosis or uterine fibroids, where different signaling pathways might be involved. Moreover, their research tends to emphasize trophoblast cell attachment during implantation rather than the invasiveness associated with EMT in endometrial cells, which was the focus of our study.

EMT has been proposed as a pivotal factor in the pathogenesis of endometriosis. In ectopic endometriotic lesions, EMT-specific pathways include those involving Twist, Snail, Slug, zinc finger E-box-binding homeobox 1/2 (ZEB1/2), E/N-cadherin, keratins, and claudins [36]. In most studies, reduced E-cad expression and increased N-cad expression in ectopic endometriotic lesions significantly differed between ectopic and eutopic endometria [37,38,39]. Unlike endometriotic lesions, eutopic endometria does not exhibit many EMT properties. Instead, the endometrium exhibited mild EMT resistance during the mid-secretory phase, the preparatory period for implantation, due to the high E-cad expression and low N-cad expression observed in this study.

The mechanism of cadherin changes within the eutopic endometrium in infertility-related disease remains largely unknown. During the WOI, which occurs in the mid-secretory phase, endometrial receptivity relies on molecular processes that are regulated by hormones [25]. Dysregulation of hormone-responsive signaling, such as the up-regulation of estrogen-induced cell proliferation, inflammation, and progesterone resistance, leads to changes in endometrial receptivity in women with endometriosis [40]. In particular, progesterone resistance, characterized by inflammatory reactions or a dysfunctional progesterone receptor, impacts abnormal endometrial changes during the WOI [15]. Recently, Huang et al. reported that downstream mediators of progesterone, such as Kruppel-like factor 15 (KLF15), may alter the EMT properties in infertility-related diseases [33]. They revealed that the progesterone receptor directly binds to KLF15, a transcription factor in mid-secretory epithelial endometrial cells, leading to reduced KLF15 expression in endometriosis patients due to progesterone resistance. Additionally, it was discovered that in vitro knockdown of KLF15 leads to an increase in E-cad mRNA and protein levels, while vimentin levels decrease. This progesterone resistance might be responsible for the mediating factor regulating the expression of cadherin molecules observed in our study. However, this association has not been established in uterine fibroids. It is worth noting that LIF expression is decreased in the endometria of women with fibroids [41], and since LIF can influence various cellular processes, including adhesion, through signaling pathways such as the JAK/STAT pathway [42], further research is needed to clarify this relationship.

A clinical application for measuring optimal endometrial receptivity is the endometrial receptivity array (ERA), a molecular array developed by Diaz-Gimeno et al. in 2011 to identify the optimal timing of endometrial receptivity for embryo transfer [43]. The ERA is particularly valuable for identifying the personalized WOI in women with recurrent implantation failure, where the WOI may be displaced, making successful embryo transfer challenging, rather than in women who already have a good prognosis [44]. In this context, the altered cadherin expression profiles observed in this study in cases of endometriosis and uterine fibroids could serve as biomarkers for assessing endometrial receptivity. Screening for E-cad and N-cad levels in the endometria of women with endometriosis or uterine fibroids before undergoing ART could help identify patients at risk for impaired receptivity, potentially guiding personalized treatment strategies. Furthermore, if future studies confirm that the imbalance in cadherin expression directly impairs endometrial receptivity, targeting the regulatory pathways of E-cad and N-cad expression may represent a viable therapeutic strategy to improve the implantation environment in affected women.

The strength of this prospective study is the use of human endometrium samples and the focus on the mid-secretory phase, called the WOI. However, the weakness is that the sample size was small, diagnoses were made using only imaging, and there was no pathological confirmation of ovarian endometrioma or uterine fibroids. Additionally, although we detected cadherin expression in infertility-related diseases, we did not directly link E-cad and N-cad expression to subfertility/infertility. In subsequent studies, we plan to prove E/N-cad expression in the endometrium of sub/infertile women with these diseases and compare fertility rates. Further studies on the signaling pathways of these cadherins in the endometria of patients with endometriosis and uterine fibroids are also needed.

## 5. Conclusions

A higher expression of E-cad and a lower expression of N-cad were observed in the endometria of women with endometriosis and uterine fibroids compared with those in healthy women in the mid-secretory phase. This mesenchymal–epithelial transition-like behavior of the endometrium in infertility-related diseases may be related to the difficult environment for embryo implantation through the resistance to endometrial receptivity.

## Figures and Tables

**Table 1:** Details of PCR primers used in this study.

Gene		Primers
*E-cad*	Forward	5′-CGAGAGCTACACGTTCACGG-3′
	Reverse	5′-GGGTGTCGAGGGAAAAATAGG-3′
*N-cad*	Forward	5′-AGCCAACCTTAACTGAGGAGT-3′
	Reverse	5′-GGCAAGTTGATTGGAGGGATG-3′
*β* *-actin*	Forward	5′-CTCTTCCAGCCTTCCTTCCT-3′
	Reverse	5′-AGCACTGTGTTGGCGTACAG-3′

**Table 2:** Characteristics of women with ovarian endometrioma or uterine fibroids.

Characteristics	Control Median (IQR) or n (%) (N = 6)	Ovarian Endometrioma Median (IQR) or n (%) (N = 17)	*p*-Value	Uterine Fibroids Median (IQR) or n (%) (N = 16)	*p*-Value
Age (years)	39.5 (34.8–42.0)	36.0 (30.5–41.5)	0.354	41.0 (38.3–43.8)	0.261
BMI (kg/m^2^) *	24.8 (21.8–26.2)	21.5 (19.6–24.4)	0.101	22.8 (20.6–24.0)	0.407
Nulliparity	2 (33.3)	10 (58.8)	0.371	7 (43.8)	0.100
Dysmenorrhea (NRS **)	1.5 (0–4.3)	4.0 (2.0–6.5)	0.101	2.0 (1.0–6.5)	0.329
CA125 (U/mL)		33.4 (23.0–53.4)		20.0 (13.0–24.6)	
AMH (ng/mL) ***		1.27 (0.76–2.15)		2.03 (0.72–4.14) (n = 9)	
***Ovarian endometrioma***					
Largest diameter (cm)		6.3 (3.5–7.5)			
Bilaterality		4 (23.5)			
Combined uterine fibroids		7 (41.2)			
***Uterine fibroids***					
Largest diameter (cm)				7.4 (6.1–9.1)	
Number of fibroids				2 (1–3)	
Type of largest fibroid					
Subserosal				2 (12.5)	
Intramural				13 (81.3)	
Submucosal				1 (6.3)	

* BMI, body mass index; ** NRS, numeric rating scale; *** AMH, anti-Müllerian hormone.

## Data Availability

Research data are available upon request to the corresponding author.
